# Optimization Models for Scheduling of Jobs

**DOI:** 10.6028/jres.111.009

**Published:** 2006-04-01

**Authors:** S. H. Sathish Indika, Douglas R. Shier

**Affiliations:** Department of Mathematical Sciences, Clemson University, Clemson, SC 29634-0975

**Keywords:** aircraft, bin packing, heuristic, integer programming, maintenance, optimization, scheduling

## Abstract

This work is motivated by a particular scheduling problem that is faced by logistics centers that perform aircraft maintenance and modification. Here we concentrate on a single facility (hangar) which is equipped with several work stations (bays). Specifically, a number of jobs have already been scheduled for processing at the facility; the starting times, durations, and work station assignments for these jobs are assumed to be known. We are interested in how best to schedule a number of new jobs that the facility will be processing in the near future. We first develop a mixed integer quadratic programming model (MIQP) for this problem. Since the exact solution of this MIQP formulation is time consuming, we develop a heuristic procedure, based on existing bin packing techniques. This heuristic is further enhanced by application of certain local optimality conditions.

## 1. Introduction

The present study was motivated by a problem encountered by an aircraft logistics center that houses a number of facilities (hangars), each of which contains multiple work stations (bays). The center offers its customers a wide variety of aircraft maintenance services (jobs), including structural repairs, passenger/freight conversions, and engine changes. In this paper we concentrate on a single hangar which is equipped with several bays.

It is assumed that the company has contractual obligations for a number of existing jobs, which have to be completed in the hangar. These existing jobs are characterized by their specified starting times, bay assignments, and job durations (*spans*). Thus, a given bay will be idle during the time periods between existing jobs. We are interested in how best to schedule a number of new jobs into these vacant spaces. Specifically, the company wishes to construct a manufacturing schedule that can be flexible in accommodating future incoming jobs.

First we present a mixed integer quadratic programming (MIQP) approach to model this scheduling problem. Solution of this MIQP gives a schedule that optimizes the overall facility utilization, while providing increased flexibility to accommodate future jobs. Next we develop local optimality conditions for the common types of changes that can occur to a particular schedule (job switches). These conditions enable us to better understand optimal solutions and also to conduct sensitivity analyses.

The exact MIQP formulation of the scheduling problem has *mn* binary variables, where *m* denotes the number of vacant spaces and *n* denotes the number of new jobs. Since there are 2*^mn^* possible choices for the binary variables, exact solution of our MIQP model is possible only for very small problems. Consequently we develop a heuristic procedure by considering the vacant spaces as *bins* with varying capacities and applying some modified bin packing techniques. We can also incorporate the local optimality conditions to improve this basic heuristic approach.

## 2. Optimization Model

**Fig. 1:**
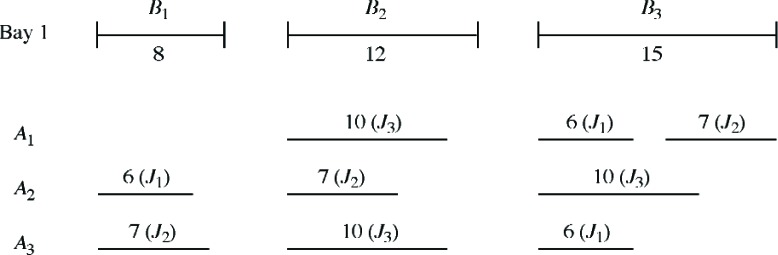
Assignments *A*_1_, *A*_2_, *A*_3_.

First we consider a very simplified scenario in order to motivate our optimization model. Suppose there is only one bay and, relative to the jobs already scheduled for that bay, there are three vacant spaces (bins) *B*_1_, *B*_2_, *B*_3_ of lengths (capacities) 8 d, 12 d, 15 d. Also suppose that three new jobs *J*_1_, *J*_2_, *J*_3_ with spans of 6 d, 7 d, 10 d are to be assigned to the bay. Several ways of assigning the three new jobs to the three bins are depicted in Fig. 1. Intuitively, it seems that assignment *A*_3_ is the most desirable of the three, because it provides the largest contiguous space remaining in a bin (9 d) after assigning the three jobs. For example, by adopting assignment *A*_3_, the center is capable of handling a future job that requires a span of 9 d or handling two additional jobs with spans of 6 d and 3 d, whereas the other two assignments cannot. Here we observe that creating a large amount of space in a bin (or in a few bins) after assigning the jobs is generally preferred over small amounts of space that are scattered over many bins.

To be more precise, let us define the *residual capacity* of a bin as the capacity that remains after assigning a job (or jobs) to that particular bin. Since the sum of the residuals is fixed (in this example totaling 12) for all assignments, we consider the sum of squared residuals for these assignments. Assignment *A*_1_ has residual capacities 8, 2, 2 with sum of squares 64 + 4 + 4 = 72; similarly, *A*_2_ has residual capacities 2, 5, 5 with sum of squares 54, while *A*_3_ has residual capacities 1, 2, 9 with sum of squares 86. We observe that assignment *A*_3_ has the largest sum of squares, as a result of the contribution from the highest residual capacity term. In general, we argue that an assignment with the largest sum of squares should be preferred because it leaves a higher residual capacity in a bin (or bins) instead of smaller residual capacities that are scattered over several bins. In turn this gives added flexibility to the company in accommodating future incoming jobs. We are thus motivated to use the maximization of the sum of squared residuals as our objective criterion. Since the sum of the residuals is fixed, we are equivalently maximizing the variance of the residuals.

### 2.1 Formulation

To formulate this problem, suppose that *n* new jobs are to be scheduled using *m* existing bins, with *c_k_* being the capacity of bin *B_k_*. Also suppose that job *J_i_* has span *s_i_*. Define the binary variable *x_ik_* = 1 if job *J_i_* is assigned to bin *B_k_*, and *x_ik_* = 0 otherwise. Also let *z_k_* denote the residual capacity of bin *B_k_*. Then our optimization model for the scheduling problem has the form:

$$\begin{array}{c} \max z=\sum_{k=1}^{m}z_{k}^{2}\\ \sum_{i=1}^{n}s_{i}x_{ik}+z_{k}=c_{k},\;k=1,\ldots ,m\\ \sum_{k=1}^{m}x_{ik}=1,\;i=1,\ldots ,n\\ x_{ik}\in \{0,1\},\;z_{k}\geq 0. \end{array}$$

The above optimization model has *m* + *n* equality constraints involving *mn* binary variables and *m* continuous variables. The first *m* equality constraints define the residual capacity variables *z_k_*; the nonnegativity of *z_k_* ensures that the set of jobs assigned to bin *B_k_* should not exceed that bin’s capacity *c_k_*. The remaining *n* equality constraints require that each job should be assigned to exactly one bin. Since we are optimizing a quadratic objective function over the (linear) constraint set, this model can be classified as a mixed integer quadratic programming (MIQP) problem. Since the objective function is a sum of squares, this problem is a type of convex maximization problem, a very difficult type of mixed integer optimization problem.

Thus far we have not considered the starting time *t_k_* associated with each bin. We can easily incorporate such starting times into our MIQP model by simply modifying the objective function:

$$\max z(\beta )=\sum_{k=1}^{m}{\left(z_{k}+\beta t_{k}\right)}^{2},$$ (1)

where *β* is a nonnegative valued parameter. When *β* = 0, we recover our original model in which maximizing the sum of squared residuals (“good fit”) is the sole criterion. However, by increasing the value of *β*, we emphasize the contribution of the starting time and thus encourage new jobs to be assigned earlier in time. The parameter *β* can be interpreted as the increased importance that the decision maker would like to give to earlier completion of jobs. It is not hard to verify that z(*β*) is a piecewise quadratic, increasing convex function of *β*.

The above model can be easily applied to situations in which there are multiple bays within a hangar. Indeed for the purposes of the optimization model MIQP, it is only necessary to maintain a list of bins with their capacities and starting times, without regard to the particular bay associated with each bin. In the multiple bay context, the parameter *β* can still be interpreted as the relative importance of a good fit versus an assignment of jobs to bins occurring earlier in time.

### 2.2 Examples

**Fig. 2:**
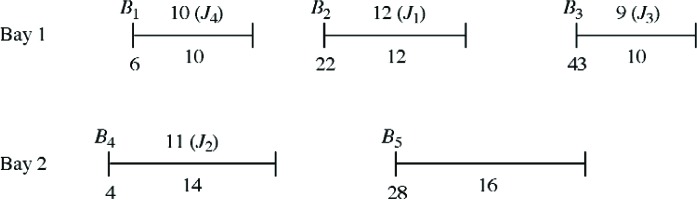
Assignment *A*_1_, optimal for 0 ≤ *β* ≤ 0.11.

In order to get some insights into the proposed MIQP model we present two numerical examples. We have used the global optimization package LINGO [4] to solve these MIQP problems exactly. *Example 1* involves *m* = 5 bins and two bays: the first three bins *B*_1_, *B*_2_, *B*_3_ are associated with bay 1 and the other two bins *B*_4_, *B*_5_ with bay 2. Capacities and starting times for these bins are given in Table 1. Four new jobs *J*_1_, *J*_2_, *J*_3_, *J*_4_ are to be assigned; these jobs have the span times *s_i_* given in Table 2. Using LINGO we obtained the exact solution of the MIQP model for various values of *β*. It turned out that there were only three sets of optimal solutions as the parameter *β* was varied. For 0 ≤ *β* ≤ 0.11, the optimal assignment *A*_1_ allocates jobs *J*_1_, *J*_2_, *J*_3_, *J*_4_ to bins *B*_2_, *B*_4_, *B*_3_, *B*_1_ respectively. A schematic illustration of assignment *A*_1_ is given in Fig. 2.

For the range 0.11 ≤ *β* ≤ 0.4, the optimal assignment *A*_2_ allocates jobs *J*_1_, *J*_2_, *J*_3_, *J*_4_ to bins *B*_4_, *B*_2_, *B*_3_, *B*_1_ respectively. Thus the only modification that occurs to the optimal job schedule, in changing from assignment *A*_1_ to assignment *A*_2_, is that jobs *J*_1_, *J*_2_ interchange positions between bins *B*_2_, *B*_4_. Over the final range 0.4 ≤ *β* < ∞, the optimal assignment *A*_3_ allocates jobs *J*_1_, *J*_2_, *J*_3_, *J*_4_ to bins *B*_4_, *B*_2_, *B*_5_, *B*_1_ respectively. The only difference between assignment *A*_2_ and assignment *A*_3_ is the movement of job *J*_3_ from bin *B*_3_ to bin *B*_5_. This behavior is consistent with our earlier observation that increasing *β* favors moving jobs to bins that occur earlier in time: *B*_5_ has a starting time of 28, earlier than the starting time 43 for *B*_3_.

**Fig. 3:**
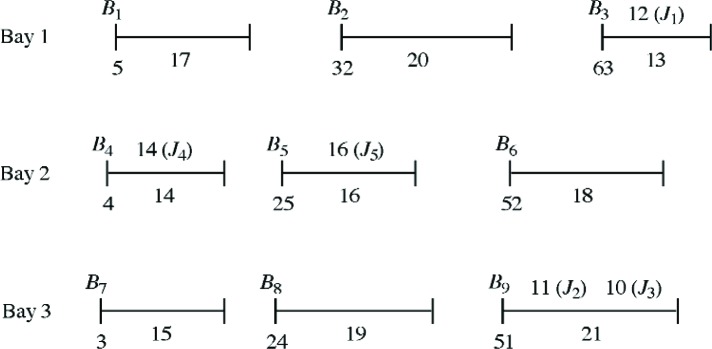
Assignment *A*_4_, optimal for 0 ≤ *β* ≤ 0.004.

*Example 2* involves *m* = 9 bins and three bays, each containing three bins. We are interested in assigning five new job *J*_1_, …., *J*_5_ to these bins. The data for this problem are given in Tables 3 and 4. Here we also investigated the changes in optimal solutions as the parameter *β* is varied. For this problem, there were eight sets of optimal solutions as the parameter *β* was varied over the ranges: [0, 0.004], [0.004, 0.033], [0.033, 0.05], [0.05, 0.079], [0.079, 0.15], [0.15, 1], [1, 3], [3, ∞]. As one illustration, Fig. 3 presents the optimal solution *A*_4_ over the first of these ranges. In assignment *A*_4_, jobs *J*_1_, *J*_4_, *J*_5_ are assigned to bins *B*_3_, *B*_4_, *B*_5_ whereas jobs *J*_2_, *J*_3_ are assigned to bin *B*_9_.

## 3. Local Optimality Conditions

In the previous section, we observed several transformations that occurred in changing from one optimal schedule to another optimal schedule. Specifically, in changing from *A*_2_ to *A*_3_, a job (*J*_3_) moves from one bin to another bin, a transformation we call a *move*. In changing from *A*_1_ to *A*_2_, two jobs (*J*_1_, *J*_2_) interchange positions between two bins, termed a *swap*. In addition, another simple transformation involves transferring jobs from several bins to a common bin (*grouping*) or vice versa (*ungrouping*). Collectively we call these types of changes, and combinations thereof, *job switches*. In this section we will first study the individual effects of each type of job switch upon a given assignment. This enables us to develop local optimality conditions for these types of job switches, relative to a given assignment. Using these local optimality conditions, it is possible to identify job switches that can improve a given feasible solution. Moreover, these local optimality conditions can aid us in carrying out a sensitivity analysis with respect to the parameter *β*.

### 3.1 Move

**Fig. 4:**
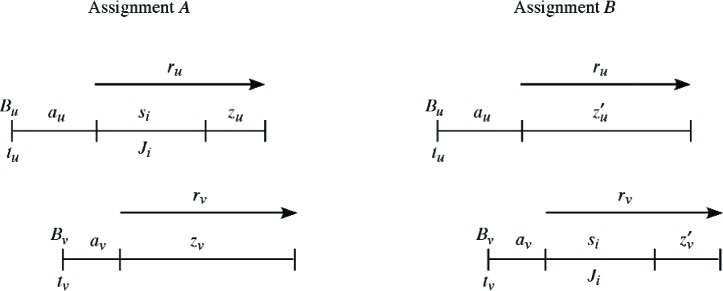
Job *J_i_* moves from bin *B_u_* to bin *B_v_*.

Consider the two assignments *A* and *B* shown in Fig. 4. In assignment *A*, job *J_i_* is currently assigned to bin *B_u_*, whereas in assignment *B*, job *J_i_* has been moved to bin *B_v_*. Otherwise the two assignments are identical. The starting times of bins *B_u_* and *B_v_* are *t_u_* and *t_v_* respectively, and the span time of job *J_i_* is *s_i_*. Define *a_u_* (resp. *a_v_*) as the capacity utilized by the other jobs that are already assigned to *B_u_* (resp. *B_v_*), and *r_u_* (resp. *r_v_*) as the remaining capacity of bin *B_u_* (resp. *B_v_*) before the assignment of job *J_i_*. The residual capacities of bins *B_u_* and *B_v_* in assignment *A* are defined as *z_u_* and *z_v_* respectively, whereas the corresponding residual capacities of bins *B_u_* and *B_v_* in assignment *B* are defined as *z_u_*′ and *z_v_*′. Thus *z_u_* = *r_u_* − *s_i_*, *z_v_* = *r_v_*, *z_u_*′ = *r_u_*, *z_v_*′ = *r_v_* − *s_i_*.

Let *Z_A_*, *Z_B_* denote the objective function values in Eq. (1) for assignments *A*, *B*. Since the only difference between the two assignments is the movement of job *J_i_* from bin *B_u_* to bin *B_v_*, we can ignore the other contributions in the objective function values *Z_A_* and *Z_B_*, writing

$$\begin{array}{l} Z_{A}={\left(z_{u}+\beta t_{u}\right)}^{2}+{\left(z_{v}+\beta t_{v}\right)}^{2}={\left(r_{u}-s_{i}+\beta t_{u}\right)}^{2}+{\left(r_{v}+\beta t_{v}\right)}^{2}\\ Z_{B}={\left({z^{\prime }}_{u}+\beta t_{u}\right)}^{2}+{\left({z^{\prime }}_{v}+\beta t_{v}\right)}^{2}={\left(r_{u}+\beta t_{u}\right)}^{2}+{\left(r_{v}-s_{i}+\beta t_{v}\right)}^{2} \end{array}$$

If we define *y_u_* = *r_u_* + *βt_u_*, *y_v_* = *r_v_* + *βt_v_*, then the above expressions simplify to

$$Z_{A}={\left(y_{u}-s_{i}\right)}^{2}+y_{v}^{2},\;Z_{B}=y_{u}^{2}+{\left(y_{v}-s_{i}\right)}^{2}.$$

Consequently ∆*Z* = *Z_A_* − *Z_B_* = 2(*y_v_* − *y_u_*)*s_i_* is the change in objective function (1). Assignment *A* will be locally optimal with respect to the move in Fig. 4 if and only if ∆*Z* ≥ 0; since *s_i_* > 0 this is equivalent to *y_v_* − *y_u_* ≥ 0. However, if *y_v_* − *y_u_* < 0 then it is beneficial to move job *J_i_* from bin *B_u_* to bin *B_v_*, assuming this move is feasible.

**Fig. 5:**
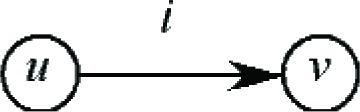
Graphical representation of a move.

It will be convenient to use an alternative representation to depict the move shown in Fig. 4. Namely, we denote the bins *B_u_* and *B_v_* by vertices *u* and *v*, and we use a directed edge *i* to denote the movement of job *J_i_* from bin *B_u_* to bin *B_v_*. This is depicted in Fig. 5.

To illustrate how this local optimality condition can be applied, consider Example 1 and assignment *A*_2_, which allocates jobs *J*_1_, *J*_2_, *J*_3_, *J*_4_ to bins *B*_4_, *B*_2_, *B*_3_, *B*_1_. The only feasible moves involve transferring a job from one of the bins *B*_1_,…, *B*_4_ to bin *B*_5_. For example, assignment *A*_2_ remains locally optimal for moving job *J*_3_ from bin *B*_3_ to *B*_5_ if and only if 0 ≤ *y*_5_ − *y*_3_ = (16 + 28*β*) − (10 + 43 *β*) = 6 − 15 *β*, so that *β* ≤ 0.4. The other conditions 0 ≤ *y*_5_ − *y*_1_, 0 ≤ *y*_5_ − *y*_2_, 0 ≤ *y*_5_ − *y*_4_ hold automatically since *β* ≥ 0. The result is that assignment *A*_2_ is locally optimal under moves for all *β* ≤ 0.4, consistent with the results obtained earlier from LINGO.

### 3.2 Swap

**Fig. 6:**
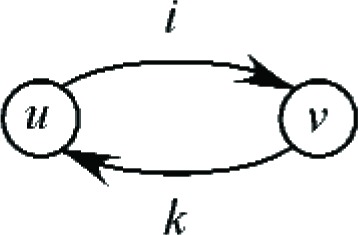
A swap of jobs *J_i_* and *J_k_*.

We now consider the swapping of jobs, relative to two assignments *A* and *B*. In assignment *A*, jobs *J_i_* and *J_k_* are assigned to bins *B_u_* and *B_v_* respectively, whereas in assignment *B*, jobs *J_i_* and *J_k_* are assigned to bins *B_v_* and *B_u_*. Therefore the only difference between the two assignments is that jobs *J_i_* and *J_k_* are swapped between bins *B_u_* and *B_v_*. This is illustrated in Fig. 6. Again define *a_u_* (resp. *a_v_*) as the capacity utilized by the other jobs that are assigned to *B_u_* (resp. *B_v_*), and *r_u_* (resp. *r_v_*) as the remaining capacity of bin *B_u_* (resp. *B_v_*) before the assignment of jobs *J_i_* and *J_k_*. The residual capacities of bins *B_u_* and *B_v_* in assignment *A* are defined as *z_u_* and *z_v_* respectively, whereas the corresponding residual capacities of bins *B_u_* and *B_v_* in assignment *B* are defined as *z_u_*′ and *z_v_*′. Thus *z_u_* = *r_u_* − *s_i_*, *z_v_* = *r_v_* − *s_k_*, *z_u_*′ = *r_u_* − *s_k_*, *z_v_*′ = *r_v_* − *s_i_*.

As before, let *Z_A_*, *Z_B_* be the objective function values for assignments *A*, *B*. Since the only difference between the two assignments is the swapping of jobs *J_i_* and *J_k_* between bins *B_u_* and *B_v_*, we can ignore the other contributions in the objective function values *Z_A_* and *Z_B_*, writing

$$\begin{array}{l} Z_{A}={\left(z_{u}+\beta t_{u}\right)}^{2}+{\left(z_{v}+\beta t_{v}\right)}^{2}={\left(r_{u}-s_{i}+\beta t_{u}\right)}^{2}+{\left(r_{v}-s_{k}+\beta t_{v}\right)}^{2}={\left(y_{u}-s_{i}\right)}^{2}+{\left(y_{v}-s_{k}\right)}^{2}\\ Z_{B}={\left({z^{\prime }}_{u}+\beta t_{u}\right)}^{2}+{\left({z^{\prime }}_{v}+\beta t_{v}\right)}^{2}={\left(r_{u}-s_{k}+\beta t_{u}\right)}^{2}+{\left(r_{v}-s_{i}+\beta t_{v}\right)}^{2}={\left(y_{u}-s_{k}\right)}^{2}+{\left(y_{v}-s_{i}\right)}^{2} \end{array}$$

Therefore ∆*Z* = *Z_A_* − *Z_B_* = 2(*y_v_* − *y_u_*)*s_i_* + 2(*y_u_* − *y_v_*)*s_k_* = 2(*y_v_* − *y_u_*)(*s_i_* − *s_k_*) and assignment *A* is locally optimal with respect to the indicated swap if and only if ∆*Z* ≥ 0. Otherwise it is beneficial to swap jobs *J_i_* and *J_k_*, assuming that such a swap is feasible.

By way of illustration, consider assignment *A*_1_ of Example 1, shown in Fig. 2. The only feasible swaps involve exchanging jobs *J*_1_ and *J*_2_ or exchanging jobs *J*_3_ and *J*_4_. Assignment *A*_1_ remains locally optimal for swapping job *J*_1_ in bin *B*_2_ with job *J*_2_ in *B*_4_ if and only if (*y*_4_ − *y*_2_)(*s*_1_ − *s*_2_) ≥ 0. Simplifying produces 0 ≤ [(14 + 4*β*) − (12 + 22*β*)](12 − 11) = 2 − 18*β* or $\beta \leq \frac{1}{9}$. Swapping jobs *J*_3_ and *J*_4_ between bins *B*_3_ and *B*_1_ imposes the condition (*y*_3_ − *y*_1_)(*s*_4_ − *s*_3_) ≥ 0 which simplifies to 37*β* ≥ 0, which clearly holds for *β* ≥ 0. In summary, assignment *A*_1_ is locally optimal for swaps over the range $0\leq \beta \leq \frac{1}{9}$, consistent with the results obtained earlier from LINGO.

### 3.3 Grouping and Ungrouping

**Fig. 7:**
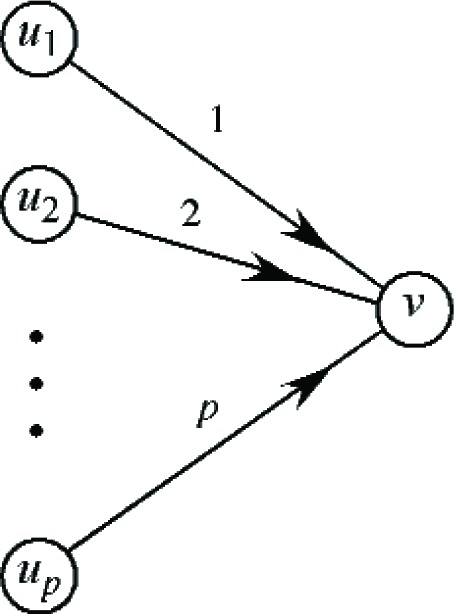
Grouping of jobs *J*_1_, …, *J_p_*.

Another simple job switch involves the grouping of jobs, in which *p* jobs are moved from separate bins to a new bin. Specifically, suppose that in assignment *A*, jobs *J*_1_, …, *J_p_* are assigned to bins $B_{u_{1}},\ldots ,B_{u_{p}}$, whereas in assignment *B*, they are all grouped together in the single bin *B_v_*. This transformation is illustrated in Fig. 7. Here we can define the utilized capacity of all bins after the assignment of jobs other than *J*_1_, …, *J_p_*, as well as the residual capacities of bins $B_{u_{1}},\ldots ,B_{u_{p}}$, in a manner similar to that done previously.

The associated objective function values for these assignments are given by

$$\begin{array}{l} Z_{A}=\sum_{i=1}^{p}{\left(z_{u_{i}}+\beta t_{u_{i}}\right)}^{2}+{\left(z_{v}+\beta t_{v}\right)}^{2}=\sum_{i=1}^{p}{\left(r_{u_{i}}-s_{i}+\beta t_{u_{i}}\right)}^{2}+{\left(r_{v}+\beta t_{v}\right)}^{2}\\ Z_{B}=\sum_{i=1}^{p}{\left({z^{\prime }}_{u_{i}}+\beta t_{u_{i}}\right)}^{2}+{\left({z^{\prime }}_{v}+\beta t_{v}\right)}^{2}=\sum_{i=1}^{p}{\left(r_{u_{i}}+\beta t_{u_{i}}\right)}^{2}+{\left(r_{v}-\sum_{i=1}^{p}s_{i}+\beta t_{v}\right)}^{2} \end{array}$$

Simplification then produces

$$\Delta Z=Z_{A}-Z_{B}=2\left[\sum_{i=1}^{p}(y_{v}-y_{u_{i}})s_{i}-\sum_{i<j}s_{i}s_{j}\right].$$

An analogous development produces the following expression when *p* jobs *J*_1_, …, *J_p_* are moved from a single bin *B_u_* to *p* separate bins $B_{v_{1}},\;\ldots ,B_{v_{p}}$.

$$\Delta Z=Z_{A}-Z_{B}=2\left[\sum_{i=1}^{p}(y_{v_{i}}-y_{u})s_{i}+\sum_{i<j}s_{i}s_{j}\right].$$

Notice the change in sign in the last summation from negative to positive for the ungrouping ∆*Z*, compared with the grouping ∆*Z*.

To illustrate the ungrouping of jobs, consider assignment *A*_4_ in Example 2, shown in Fig. 3. Suppose that jobs *J*_2_, *J*_3_ are moved from bin *B*_9_ to bins *B*_7_, *B*_1_ respectively. (This is a feasible ungrouping of jobs.) Then

$$\begin{array}{l} \Delta Z=2[(y_{7}-y_{9})s_{2}+(y_{1}-y_{9})s_{3}+s_{2}s_{3}]\\ \;=2([(15+3\beta )-(21+51\beta )]11+[(17+5\beta )\\ \;-(21+51\beta )]10+11\cdot 10)=2(4-988\beta ). \end{array}$$

Consequently, the proposed ungrouping will be advantageous when ∆*Z* < 0 or $\beta >\frac{1}{247}$.

### 3.4 More Complex Job Switches

**Fig. 8:**
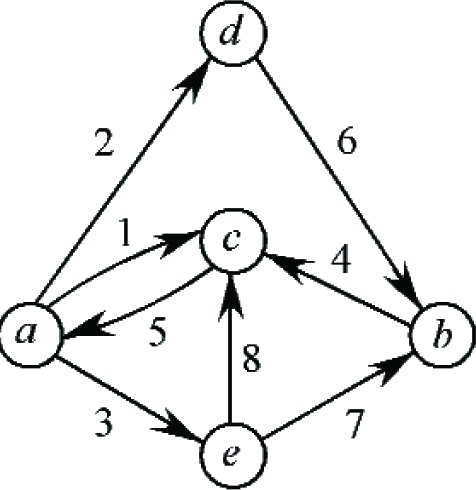
Graphical representation of a more complicated scenario.

This section considers combining the previous transformations (moves, swaps, groupings, and ungroupings) into more complex job switches. To motivate the general case, we first consider an example involving eight jobs and five bins. Namely, the transformations that occur, in changing from the current assignment *A* to the new assignment *B* are as follows: jobs *J*_1_, *J*_5_ swap between bins *B_a_*, *B_c_*; jobs *J*_2_, *J*_3_ ungroup from bin *B_a_* to bins *B_d_*, *B_e_*; job *J*_4_ moves from *B_b_* to *B_c_* while job *J*_6_ moves from *B_d_* to *B_b_*; and jobs *J*_7_, *J*_8_ ungroup from *B_e_* to *B_b_*, *B_c_*. See Fig. 8 for a graphical representation of this more complex rearrangement. Following the previous development, we obtain

$$\begin{array}{l} Z_{A}={\left(y_{a}-s_{1}-s_{2}-s_{3}\right)}^{2}+{\left(y_{b}-s_{4}\right)}^{2}+{\left(y_{c}-s_{5}\right)}^{2}+{\left(y_{d}-s_{6}\right)}^{2}+{\left(y_{e}-s_{7}-s_{8}\right)}^{2}\\ Z_{B}={\left(y_{a}-s_{5}\right)}^{2}+{\left(y_{b}-s_{6}-s_{7}\right)}^{2}+{\left(y_{c}-s_{1}-s_{4}-s_{8}\right)}^{2}+{\left(y_{d}-s_{2}\right)}^{2}+{\left(y_{e}-s_{3}\right)}^{2}\\ \Delta Z=2[\left(y_{c}-y_{a}\right)s_{1}+(y_{d}-y_{a})s_{2}+(y_{e}-y_{a})s_{3}+(y_{c}-y_{b})s_{4}\\ +(y_{a}-y_{c})s_{5}+(y_{b}-y_{d})s_{6}+(y_{b}-y_{e})s_{7}+(y_{c}-y_{e})s_{8}+s_{1}s_{2}\\ +s_{2}s_{3}+s_{1}s_{3}+s_{7}s_{8}-s_{1}s_{4}-s_{4}s_{8}-s_{1}s_{8}-s_{6}s_{7}]. \end{array}$$

Notice that for each directed edge (*i*, *j*) representing the movement of job *J_k_* in Fig. 8 there is a term 2(*y_j_* − *y_i_*)*s_k_* in ∆*Z*. There are also terms in ∆*Z* to represent the ungrouping and grouping of jobs. Jobs *J*_1_, *J*_2_, *J*_3_ ungroup at vertex a, giving rise to the product terms *s*_1_*s*_2_ + *s*_2_*s*_3_ + *s*_1_*s*_3_; likewise the ungrouping of jobs *J*_7_, *J*_8_ at vertex *e* gives the term *s*_7_*s*_8_. On the other hand, jobs *J*_1_, *J*_4_, *J*_8_ group at vertex *c*, producing the (negative) term −(*s*_1_*s*_4_ + *s*_4_*s*_8_ + *s*_1_*s*_8_), while jobs *J*_6_, *J*_7_ group at vertex *b*, giving −*s*_6_*s*_7_.

In general suppose that the directed graph *G* = (*V*, *E*) represents the specified changes to a current assignment, where the set *V* of vertices represents the bins and the set *E* of directed edges represents the movement of jobs between bins. Denote the span time of the job associated with edge (*i*, *j*) by *s_ij_*. Also let Γ^+^(*i*) denote the set of edges leaving vertex *i* and let Γ^−^(*i*) denote the set of edges entering vertex *i*. Then the expression for ∆*Z* becomes

$$\begin{array}{l} \Delta Z=2\sum_{(i,j)\in E}(y_{j}-y_{i})s_{ij}+\sum_{i\in V}\sum \left\{s_{a}s_{b}:a,b\in \Gamma^{+}(i),a\neq b\right\}\\ -\sum_{i\in V}\sum \left\{s_{a}s_{b}:a,b\in \Gamma^{-}(i),a\neq b\right\}. \end{array}$$

## 4. Heuristic Procedures

In Sec. 2.1, we modeled the scheduling problem as an MIQP with *mn* binary variables and *m* continuous variables, where *m* is the number of bins and *n* is the number of new jobs. Since the number of possible choices 2*^mn^* for the binary variables rapidly becomes large, even for small *m* and *n*, the exact mathematical solution of the MIQP model is very time consuming. Therefore we consider heuristic solution approaches in this section, rather than exact procedures.

As noted earlier the vacant time interval between any two existing jobs can be considered a bin. Since the intervals between existing jobs are of different length, the corresponding bins have variable size. The underlying problem is then to assign the new jobs to these bins in such a way that the set of new jobs assigned to a bin fits within that bin’s capacity; this is to be done in an “optimal” fashion. Thus our scheduling problem can be viewed as a variable-sized bin packing problem [3,5] with an unusual type of objective function. In this section we will first develop a simple heuristic procedure based on existing bin packing techniques. Next we will apply the local optimality conditions developed in Sec. 3 to improve this bin packing heuristic.

### 4.1 Bin Packing Heuristic

We begin by reviewing two bin packing algorithms that are well known in the literature [2]. They are the First Fit (FF) algorithm and the Best Fit (BF) algorithm. The objective of such standard bin packing algorithms is to minimize the number of bins that are needed to pack a given set of items. The FF algorithm assigns the next job into the lowest indexed bin into which it will fit. If the next job does not fit into any existing bin then we open a new bin and place the next job in the new bin. The BF algorithm is similar to the FF algorithm, except that it assigns the next job into that bin which will leave the smallest residual capacity after the assignment.

In our heuristic algorithm we first order the jobs in order of nonincreasing span times. There is an intuitive appeal to ordering the span times in this manner: we assign the jobs with higher span times first and hope that we can accommodate the jobs with smaller span times using the spaces that remain. A similar strategy is adopted in existing bin packing algorithms [1,6]. Before assigning the next job (in order of nonincreasing span) to a bin, we first identify the bins into which the job can fit. Among these candidate bins, we select a bin, say bin *B_u_*, having the minimum value of *z_u_* + *βt_u_*. As shown in the proposition below, this procedure enables us to (locally) improve the current solution as much as possible at the next step. Next we assign the new job to the selected bin and update the residual capacity of that bin. We follow this procedure until all jobs have been assigned. We now summarize more formally the steps of this heuristic algorithm.

bin_packing

1. Order the jobs by nonincreasing span time.
2. For the next job *J_k_* in order, with span *s_k_*, consider only those bins with residual capacity at least *s_k_*. Among such bins, select bin *B_u_* to minimize *z_u_* + *βt_u_*.
3. Assign job *J_k_* to bin *B_u_* and update *z_u_* ← *z_u_* − *s_k_*.
4. If there are additional jobs to be processed, go to Step 2.

Here *β* ≥ 0 is the same parameter introduced in Sec. 2.1. When *β* = 0 we assign the next job *J_k_* into a bin (into which it fits) having the minimum current residual capacity. Since this will also leave the smallest residual capacity after the assignment, our heuristic algorithm is similar to the BF algorithm for *β* = 0. When *β* is large, we assign jobs to bins in order of increasing starting time. In other words, jobs are assigned to bins that occur earliest in time. This is analogous to assigning a job to the lowest indexed bin into which it will fit, when bins are ordered by starting times. So when *β* is large, our heuristic algorithm behaves similar to the FF algorithm. In this way we have blended both BF and FF into our particular bin packing heuristic. We now demon strate that our heuristic performs the locally “best” assignment for the current job at each step of the algorithm.

**Proposition.** *The bin packing heuristic locally improves the objective function by as much as possible at the next step.*

**Proof.** Suppose that job *J_k_* with span *s_k_* is to be assigned and that bins *B*_1_, …, *B_p_* are the bins into which *J_k_* can fit. Let *z*_1_, …, *z_p_* be the current residual capacities of these bins. Select bin *B_u_* such that

$$z_{u}+\beta t_{u}=\min\{z_{1}+\beta t_{1},\ldots ,z_{p}+\beta t_{p}\}.$$ (2)

Let *Z_j_* be the objective function value (1) obtained by assigning job *J_k_* to bin *B_j_* at the next step. We claim that *Z_u_* ≥ *Z_j_* for all 1 ≤ *j* ≤ *p*. Let α denote the contribution to the objective function from all bins other than *B_u_* and *B_j_* in the current assignment. Thus

$$\begin{array}{l} Z_{u}=\alpha +{\left(z_{u}-s_{k}+\beta t_{u}\right)}^{2}+{\left(z_{j}+\beta t_{j}\right)}^{2}\\ Z_{j}=\alpha +{\left(z_{u}+\beta t_{u}\right)}^{2}+{\left(z_{j}-s_{k}+\beta t_{j}\right)}^{2} \end{array}$$

giving

$$\begin{array}{l} Z_{u}-Z_{j}=-2z_{u}s_{k}-2\beta t_{u}s_{k}+2z_{j}s_{k}+2\beta t_{j}s_{k}\\ \;=2[(z_{j}+\beta t_{j})-(z_{u}+\beta t_{u})]s_{k}\geq 0, \end{array}$$

where the final inequality follows from Eq. (2). □

We illustrate the bin packing heuristic using Example 1 when *β* = 0.3. Ordering the four jobs by nonincreasing span times places them in the sequence *J*_1_, *J*_2_, *J*_4_, *J*_3_. To begin, job *J*_1_ can fit into bins *B*_2_, *B*_4_, *B*_5_, which have residual capacities 12, 14, 16 and starting times 22, 4, 28. Since min{12 + 0.3(22), 14 + 0.3(4), 16 + 0.3(28)} = 15.2 is achieved for bin *B*_4_, we assign job *J*_1_ to bin *B*_4_ and update the residual capacity of bin *B*_4_ to *z*_4_ = 2.

We next select (in order) job *J*_2_, which can fit into bins *B*_2_, *B*_5_, with residual capacities 12, 16. Since min{12 + 0.3(22), 16 + 0.3(28)} = 18.6 is achieved for bin *B*_2_, we assign job *J*_2_ to bin *B*_2_ and update the residual capacity of bin *B*_2_ to *z*_2_ = 1. Continuing in this fashion, job *J*_4_ is assigned to bin *B*_1_ and job *J*_3_ is assigned to bin *B*_3_. Thus the heuristic assigns jobs *J*_1_, *J*_2_, *J*_3_, *J*_4_ to bins *B*_4_, *B*_2_, *B*_3_, *B*_1_ respectively. This assignment is identical to the optimal assignment *A*_2_ found for the range 0.11 ≤ *β* ≤ 0.4. In fact for Example 1 the heuristic produces optimal assignments over all three ranges for the parameter *β*.

For Example 2 the MIQP model gives eight optimal assignments corresponding to eight ranges of the parameter *β*. The assignments obtained by the heuristic procedure are identical to the optimal assignments obtained by the MIQP model in seven of these eight ranges. The only difference occurs over the range 0 ≤ *β* ≤ 0.004 for which the optimal assignment *A*_4_ is shown in Fig. 3; it assigns jobs *J*_1_, …, *J*_5_ to bins *B*_3_, *B*_9_, *B*_9_, *B*_4_, *B*_5_. By contrast, the heuristic procedure obtains a different assignment *A*_4_′, in which *J*_1_, …, *J*_5_ are assigned to *B*_3_, *B*_7_, *B*_1_, *B*_4_, *B*_5_. If however we perform in *A*_4_′ a grouping of jobs *J*_2_, *J*_3_ from bins *B*_7_, *B*_1_ to bin *B*_9_, then the change in objective function value is

$$\Delta Z=2[(y_{9}-y_{7})s_{2}+(y_{9}-y_{1})s_{3}-s_{2}s_{3}]=2(-4+988\beta ).$$

For 0 ≤ *β* < 0.004, the term in parentheses above is negative so that it is advantageous to perform this grouping. In other words, first applying the heuristic to obtain *A*_4_′ and then using an improving step (grouping) does indeed yield the optimal assignment *A*_4_ over the range.

To summarize, for Example 1 the heuristic procedure obtained the optimal solution for all ranges. In Example 2, there was one instance in which the heuristic gave a suboptimal solution. However in this case, a single job switch (grouping) was sufficient to produce the optimal solution. This encouraging success suggests a hybrid heuristic procedure that first carries out the bin packing algorithm, followed by local improvements using selected job switches. In particular, it is straightforward to check for improving moves and swaps; it is a bit more tedious to evaluate all groupings and ungroupings relative to a given assignment.

## 5. Conclusions

In this paper we have considered the scheduling of different types of aircraft maintenance programs. In this initial study, we concentrated on the simplified case where there is only a single hangar. First we developed an MIQP to model this scheduling problem. The MIQP model incorporates a parameter *β* that reflects the relative importance of a good fit versus assigning of jobs to bins occurring earlier in time. For small test problems, it is possible to obtain an exact schedule by solving the MIQP model. Results from these test problems indicated that there were relatively few optimal schedules over the range of all possible values of the specified parameter *β.* We also developed local optimality conditions for certain types of job switches, relative to a given assignment. The local optimality conditions enable us to improve a given feasible job schedule. Because exact solution of the MIQP is limited to fairly small instances, we developed a simplified heuristic procedure based on existing bin packing techniques. An area for future research is to combine the bin packing heuristic with the intelligent application of the local optimality conditions.

## Figures and Tables

**Table 1:** Bin capacities and starting times for Example 1

	bin *B_k_*				
*k*	1	2	3	4	5
*c_k_*	10	12	10	14	16
*t_k_*	6	22	43	4	28

**Table 2:** Span times of new jobs for Example 1

	job *J_i_*			
*i*	1	2	3	4
*s_i_*	12	11	9	10

**Table 3:** Bin capacities and starting times for Example 2

	bin *B_k_*								
*k*	1	2	3	4	5	6	7	8	9
*c_k_*	17	20	13	14	16	18	15	19	21
*t_k_*	5	32	63	4	25	52	3	24	51

**Table 4:** Span times of new jobs for Example 2

	job *J_i_*				
*i*	1	2	3	4	5
*s_i_*	12	11	10	14	16
